# Bmoo FIBMP-I: A New Fibrinogenolytic Metalloproteinase from *Bothrops moojeni* Snake Venom

**DOI:** 10.5402/2012/673941

**Published:** 2012-11-04

**Authors:** F. S. Torres, B. Rates, M. T. R. Gomes, C. E. Salas, A. M. C. Pimenta, F. Oliveira, M. M. Santoro, M. E. de Lima

**Affiliations:** ^1^Laboratório de Venenos e Toxinas Animais, Departamento de Bioquímica e Imunologia, Instituto de Ciências Biológicas, Universidade Federal de Minas Gerais, 31270-901 Belo Horizonte, MG, Brazil; ^2^Laboratório de Biologia Molecular de Produtos Naturais, Departamento de Bioquímica e Imunologia, Instituto de Ciências Biológicas, Universidade Federal de Minas Gerais, 31270-901 Belo Horizonte, MG, Brazil; ^3^Departamento de Ciências Fisiológicas, Instituto de Ciências Biomédicas, Universidade Federal de Uberlândia, 38400-902 Uberlândia, MG, Brazil; ^4^Instituto Nacional de Ciência e Tecnologia em Nano-Biofarmacêutica (N-Biofar), Belo Horizonte, MG, Brazil; ^5^Laboratório de Físico-Química de Proteínas e Enzimologia, Departamento de Bioquímica e Imunologia, Instituto de Ciências Biológicas, Universidade Federal de Minas Gerais, 31270-901 Belo Horizonte, MG, Brazil

## Abstract

A new fibrinogenolytic metalloproteinase (Bmoo FIBMP-I) was purified from *Bothrops moojeni* snake venom. This enzyme was isolated through a combination of three chromatographic steps (ion-exchange, molecular exclusion, and affinity chromatography). Analyses by reverse phase chromatography, followed by mass spectrometry, showed the presence of enzyme isoforms with average molecular mass of 22.8 kDa. The SDS-PAGE analyses showed a single chain of 27.6 kDa, in the presence and absence of reducing agent. The protein has a blocked N-terminal. One of the peptides obtained by enzymatic digestion of a reduced and S-alkylated isoform was completely sequenced by mass spectrometry (MS/MS). Bmoo FIBMP-I showed similarity with hemorrhagic factor and several metalloproteinases (MP). This enzyme degraded A*α*-chain faster than the B*β*-chain and did not affect the *γ*-chain of bovine fibrinogen. The absence of proteolytic activity after treatment with EDTA, together with the observed molecular mass, led us to suggest that Bmoo FIBMP-I is a member of the P-I class of the snake venom MP family. Bmoo FIBMP-I showed pH-dependent proteolytic activity on azocasein, but was devoid of coagulant, defibrinating, or hemorrhagic activities. The kinetic parameters of proteolytic activity in azocasein were determined (*V*_max_ = 0.4596 Uh^−1^nmol^−1^ ± 0.1031 and *K*_*m*_ = 14.59 mg/mL ± 4.610).

## 1. Introduction

 Snake venoms are complex mixtures of proteins and peptides with diverse pharmacological activities. A number of these proteins have direct action on vessels walls, platelet function, fibrinogen, and other factors of the hemostatic system [1, 2]. While neurotoxicity is the most pronounced effect of envenomation by snakes from the *Elapidae* family, envenomation by snakes from the *Viperidae* family is usually characterized by local and, in severe cases, systemic effects [3, 4]. These snake venoms are rich sources of metalloproteinases (MPs) which, along with the transmembrane ADAMS (desintegrin-like and metalloproteinase-containing proteins), are members of the reprolysins subfamily M12-family of metalloproteinases. (Snake venom metalloproteinases) SVMPs have been classified into three basic structural classes, P-I to P-III [5–7]. These metalloproteinases are synthesized in the venom gland as large multidomain proteins, including a proenzyme domain and a highly conserved zinc-proteinase domain (HEBXHXBGBXH) [6, 8, 9].

The P-I (PIa) metalloproteinase class includes proteins with molecular masses between 20–30 kDa that have low or no hemorrhagic effect, but strong direct-acting fibrino(geno)lytic activity, and contain only a metalloproteinase domain. Class P-II (P-IIa, P-IIb, P-IIc, P-IId, and P-IIe) comprises proteins with molecular masses of 30–60 kDa that contain metalloproteinase and disintegrin-like domains. Class P-III (P-IIIa, P-IIIb, P-IIIc, and P-IIId) includes a cysteine-rich domain, a melloproteinase domain, and a disintegrin-like and a lectin-like domain [2, 5, 7]. 

 Many proteinases have been purified and characterized from venoms of different *Bothrops *species such as: *Bothrops jararaca* [10–12], *Bothrops moojeni* [13–18], *Bothrops neuwiedi* [19–21], *Bothrops leucurus* [2, 22, 23], *Bothrops jararacussu* [24–26], *Bothrops asper* [27, 28], *Bothrops cotiara* [29]. 

This paper reports the isolation and partial characterization of a fibrinogenolytic metalloproteinase, Bmoo FIBMP-I, from *B. moojeni* snake venom.

## 2. Materials and Methods

### 2.1. Materials

Desiccated *B. moojeni* venom was purchased from Bioagents Serpentarium (Batatais-SP, Brazil). Acrylamide, ammonium persulfate, aprotinin, benzamidine, bromophenol blue, ethylenediaminetetracetic acid (EDTA), bovine fibrinogen,  *β*-mercaptoethanol, *N*,*N *′-methylene-bis-acrylamide, sodium dodecyl sulphate (SDS) *N*,*N*,*N *′,*N *′-tetramethylethylenediame (TEMED), glycine, Tris, molecular weight markers for electrophoresis, and all chromatographic media were purchased from Sigma Chemical Co. (St. Louis, MO, USA). All other reagents used were of analytical grade.

### 2.2. Isolation of the Metalloproteinase Bmoo FIBMP-I

The chromatographic steps were based on a previously published protocol with minor modifications [16, 30]. The crude venom of *B. moojeni* (250 mg) was dissolved in 0.05 M ammonium bicarbonate buffer (pH 7.8) and clarified by centrifugation at 10,000 g for 10 min at room temperature. The clear supernatant solution was applied to a DEAE Sephacel column (2 × 12 cm), previously equilibrated with 0.05 M ammonium bicarbonate buffer pH 7.8 and eluted with a concentration gradient (0.05 M–0.3 M) of the same buffer. Fractions of 3.0 mL/tube were collected and their absorbances were read at 280 nm. The fourth fraction, named D4, was pooled, lyophilized, dissolved in 0.05 M pH 7.8 ammonium bicarbonate, and applied to a Sephadex G-75 column (1 × 120 cm) previously equilibrated with the same buffer. The fibrinogenolytic fraction (D4G2) was lyophilized and applied to a column of Heparin Agarose (2 × 10 cm) previously equilibrated with 0.01 M pH 7.5 Tris-HCl buffer, containing 0.05 M CaCl_2_ and eluted with 0.01 M pH 7 Tris-HCl buffer containing 1 M NaCl. The flow rate was 40 mL/h and fractions of 2.0 mL were collected.

### 2.3. Reversed Phase HPLC of the Purified Metalloproteinase Bmoo FIBMP-I

The fraction showing fibrinogenolytic and azocaseinolytic activities, obtained in the previous step, was submitted to reverse phase system on a Source 15RPC column (0.46 cm × 25 cm), ÄKTA Explorer, Amersham-Pharmacia (Uppsala, Sweden), equilibrated with the eluent A (0.1% TFA in water Milli-Q), eluted by linear gradient (from 0 to 60% in 50 min, and from 60 to 100% in 7.5 min) of eluent B (acetonitrile, 0.1% TFA) at a flow rate of 2 mL/min, and monitored by absorbance at 280 nm. 

### 2.4. Biochemical Characterization

The protein concentration of the venom samples and fractions was determined by the method of microbiuret, as described by Itzhaki and Gill [31], using bovine serum albumin as standard. Polyacrylamide gel electrophoresis was performed in the presence of sodium dodecyl sulfate (SDS-PAGE) and carried out according to a previously described method [32] using 14% gels. Samples were pretreated under reducing conditions (SDS plus  *β*-mercaptoethanol) at 100°C for 5 min. Gels were stained with 0.1% Coomassie brilliant blue R-250 in ethanol: acetic acid (5 : 1, v/v) for 15 min and distained in 10% acetic acid : water (v/v). The molecular mass was estimated by interpolation from a linear logarithmic plot of relative molecular mass versus distance of migration. Standard molecular weight markers (Sigma) were b phosphorylase (97 kDa), bovine serum albumin (66 kDa), egg albumin (45 kDa), carbonic anhydrase (30 kDa), soybean trypsin inhibitor (20.1 kDa), and lactoalbumin (14.4 kDa). 

### 2.5. Sequence Analysis

The protein (0.3 mg) was reduced and S-alkylated as previously described [33, 34]. The sample containing the protein was dissolved in 1 mL of 0.6 M Tris-HCl buffer containing 6 M guanidine, pH 8.6, and 30 *μ*L of  *β*-mercaptoethanol (pure liquid 14.3 M), under nitrogen atmosphere, and incubated at 50°C for 5 h. Then, 20 *μ*L of 4-vinylpiridine (95%) were added and the samples were incubated at 37°C during 1.5 h. The protein was recovered by desalting on a Vydac C4 column (0.46 cm × 25 cm) using a gradient of 0 to 100% acetonitrile in 0.1% TFA at a flow rate of 2 mL/min. The proteins were detected by their absorbance at 216 nm. For digestion, the reduced/S-alkylated protein (0.2 mg) was dissolved in 100 *μ*L of 8 M urea and diluted to 900 *μ*L with 0.1 M NH_4_HCO_3_, pH 8.1, then digested with trypsin (5% w/w, enzyme/protein, for 4 h at 37°C). The obtained peptides were purified by reverse phase HPLC on a Vydac C4 column using extended (~4 h) linear gradient of 0–70% acetonitrile in 0.1% TFA. The peptides purified were then sequenced using MS/MS in MALDI-TOF/TOF MS Autoflex III (Bruker Daltonics, Germany). The primary sequences of the obtained peptides were compared to sequences of other related proteins in the SWISS-PROT/TREMBL data base using FASTA 3 and BLAST programs.

### 2.6. Enzymatic Activities

#### 2.6.1. Proteolytic Activity upon Fibrinogen

The fibrinogenolytic activity was assayed as described by Edgar and Prentice [35], partially modified. Five micrograms of the enzyme were added to 50 *μ*L of bovine fibrinogen (1.5 mg/mL), buffered at different pHs (0.02 M sodium acetate buffer, pH 3, 4, and 5; 0.02 M sodium phosphate buffer, pH 6; 0.02 M Tris-HCl buffer, pH 7, 8, and 9.0; sodium borate buffer, pH 10 and 11) at 37°C for 60 min. The reaction was stopped by addition of 25 *μ*L of 0.05 M Tris–HCl buffer, pH 8.8, containing 10% (v/v) glycerol, 10% (v/v)  *β*-mercaptoethanol, 2% (w/v) SDS, and 0.05% (w/v) bromophenol blue. The reaction products were then analyzed by 14% (w/v) SDS-PAGE. 

To determine the effect of temperature on the fibrinogenolytic activity, 5 *μ*g of enzyme (0.02 M Tris-HCl buffer pH 9.0) were incubated at different temperatures (40, 50, 60, 70, and 80°C) for 15 min before chilling in ice bath for 5 min. Then, the fibrinogenolytic activity was determined as described above.

The effect of inhibitors on the fibrinogenolytic activity was assayed after preincubation of the enzyme (5 *μ*g) dissolved in 0.25 mL of 0.05 M Tris-HCl buffer, pH 8.0, with 10 mM EDTA (metalloproteinase inhibitor) or 0.01 M aprotinin (serinoproteinase inhibitor) for 30 min at room temperature. 

#### 2.6.2. Hemorrhagic, Coagulant, and Defibrinating Activities

The possible haemorrhagic, coagulant, and defibrinating activities of Bmoo FIBMP-I were investigated. Hemorrhagic activity was assessed by the method of Kondo et al. [36]. Briefly, different doses of the purified enzyme (1–10 *μ*g), in 100 *μ*L of 0.9% NaCl were intradermally injected into the dorsal skin of male Swiss mice (18–22 g). After 24 h, the animals were anesthetized with ether and killed. Skins were removed and the areas of hemorrhage on their inner surfaces were scanned above a sheet of white paper. The area of each halo was cut out and its mass was measured in a precision scale. The hemorrhage was calculated through a previously determined correlation of the area of the white paper with its respective mass (7.5 mg/cm^2^). The minimum hemorrhagic dose (MHD) is defined as the dose of protein wich results in an hemorrhagic lesion of 1.0 cm^2^ after 24 h.

The coagulant activity of the enzyme was assayed as described by Thekston and Reid [37]. The minimum coagulant dose (MCD) is defined as the minimum amount of enzyme that clots bovine plasma (MCD-P) and/or a solution of bovine fibrinogen (2.0 mg/mL; MCD-F) in 60 seconds at 37°C. The defibrinating activity was tested by the method of Gené et al. [38], with minor modifications. The activity was assessed by intraperitoneal injection of different doses of the enzyme (20–100 *μ*g), in 100 *μ*L of saline solution (0.9% NaCl), into male Swiss mice (18–22 g), using four mice per group at each protein dose; control animals received 200 *μ*L of saline solution. One hour after, the animals were anesthetized and bled by cardiac puncture. Whole blood was placed in tubes and kept at 25–30°C until clotting occurred. The minimum defibrinating dose (MDD) was defined as the amount of venom able to prevent coagulation. 

#### 2.6.3. Proteolytic Activity on Azocasein (Azocaseinolytic Activity)

The proteolytic activity was quantified by the method of Leonardi et al. [39], with modifications. Several concentrations (20, 40, 60 and 80 *μ*g) of Bmoo FIBMP-I were added to 500 *μ*L of an azocasein solution (1 mg/mL in 0.2 M Tris-HCl buffer, pH 8.8, containing 0.004 M CaCl_2_), at 37°C for 60 min. The reaction was interrupted by the addition of 5% trichloroacetic acid (TCA) and then centrifuged (14,000 g, 10 min). The absorbance of the supernatant was read at 366 nm. One unit of proteolytic activity corresponds to an increase of 0.1 unit at A_366_. The influence of pH on proteolytic activity was also tested by preincubation of the enzyme fraction in various buffers at different pH (0.02 M sodium acetate buffer, pH 3, 4, and 5; 0.02 M sodium phosphate buffer, pH 6, 0; 0.02 M Tris-HCl buffer, pH 7, 8, and 9.0; sodium borate buffer, pH 10 and 11) at 37°C for 60 min. 

### 2.7. Kinetic Analyses

 The steady-state parameters *K*_*m*_ and *V*_max⁡_ were determined from initial rate measurements at various azocasein concentrations in 0.2 M Tris-HCl buffer, pH 8.8, containing 0.004 M CaCl_2_ at 25°C. At least two separate measurements were carried out for each azocasein concentration. Reactions were initiated by the addition of appropriate limiting amounts of enzymes and the formation of cleavage products was monitored at 366 nm. The normalized velocity was calculated using the conversion factor 1.578. This conversion factor was obtained by multiplying the number of hours (6 hours) by the molar mass of the protein (2.63 nmoL) and the number of activity units of the enzyme (every enhancement of 0.1 in the absorbance equals to 1 activity unit). The Michaelis constant *K*_*m*_ and *V*_max⁡_ were determined by analysis of Michaelis Menten constant plot of the normalized velocity as a function of substrate concentration.

### 2.8. Mass Spectrometry and Data Analyses

 ESI-Q-TOF mass spectrometry analyses were carried out using a Q-TOF Micro (Micromass, UK) equipped with an electrospray ionization source operated in the positive mode. The capillary voltage was 2.5–3.0 kV and the sample cone voltages were 30–40 V. Mass spectrometer calibrations were made by using sodium iodide in 100 to 2000 m/z. Samples diluted in 50% acetonitrile/0.1% TFA were introduced by using a syringe pump with a flow rate of 10 *μ*L/min. Original data (m/z) were treated (base line subtraction, smoothing, and centering) and transformed into a mass (Da) spectrum. Data analyses were carried out using MassLynx 4.0 software.

 Other analyses, MS and tandem MS, analysis were performed using a MALDI-TOF-TOF AutoFlex III (Bruker Daltonics) instrument in positive/reflector mode controlled by the Flex-Control software. Instrument calibration was achieved by using Peptide Calibration Standard II (Bruker Daltonics) as reference and  *α*-cyano-4-hydroxycinnamic acid as matrix. Samples were spotted to MTP AnchorChip 400/384 (Bruker Daltonics) targets using standard protocols for the dried droplet method.

 MS data analyses were performed by using the Flex-Analysis software (Bruker Daltonics). Peptide *de novo* sequencing was performed using a combination of manual and automatic data interpretation using the softwares FlexAnalysis and BioTools (Bruker Daltonics). Alternatively, MS/MS data were exported as *.txt files and then converted to the *.dta extension. Then such files were imported into *.psq files which are recognizable by the PepSeq (Micromass, UK) software which was also used for manual *de novo* sequencing. Similarity searches were performed with the obtained sequences using the Fasta3 tool against the Swiss-Prot data Bank, as previously described [40].

## 3. Results and Discussion

 The high proteolytic activity of *Bothrops* snake venoms is primarily responsible for most of the local and systemic effects observed during envenomation by these snakes [1]. This activity is caused by a variety of enzymes, for example, metalloproteinases, which are enzymes that possess a metal ion at the active site and are responsible for the bleeding that results from *Bothrops* bites [25, 41]. Thus, in this study, the interest was focused on this group of molecules.

**Figure 1:**
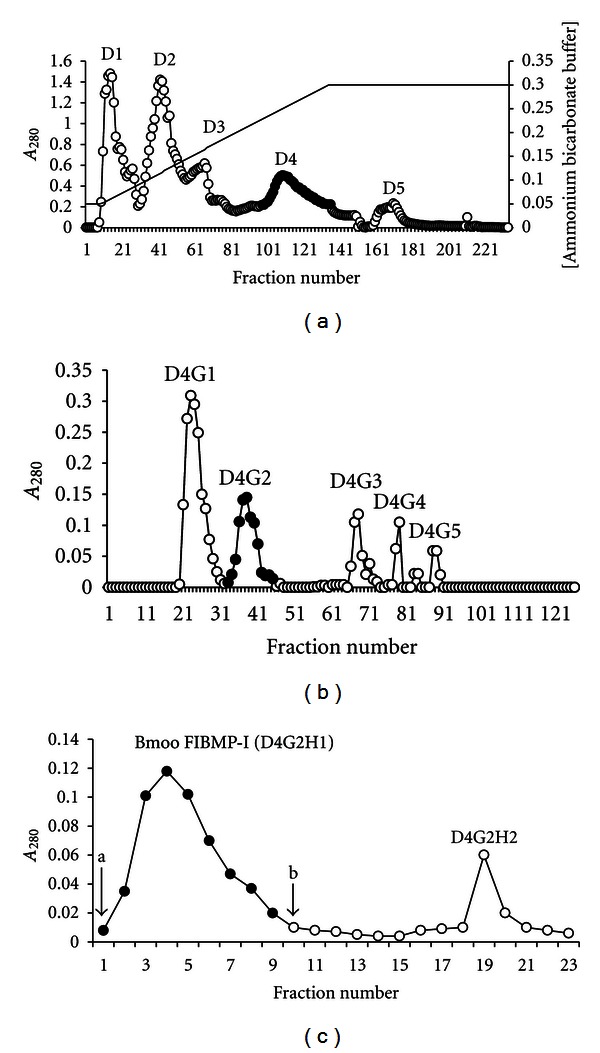
Purification of Bmoo FIBMP I from *Bothrops moojeni* venom. (a) Ion-exchange chromatography of Brazilian *B. moojeni* venom on DEAE Sephacel. The fibrinogenolytic activity was greater in fraction D4. (b) Gel filtration on Sephadex G-75 column. Fibrinogenolytic and azoproteolytic activities were observed for D4G2 fraction. (c) Affinity chromatography on Heparine Agarose resin. Fibrinogenolytic and azoproteolytic activities were observed for fraction Bmoo FIBMP-I. • Fibrinogenolytic activity (fractions pooled).

 The purification of the fibrinogenolytic enzyme Bmoo FIBMP-I from *B. moojeni* venom consisted of a three-step procedure including ion-exchange on DEAE Sephacel, gel filtration on Sephadex G-75, and affinity chromatography on Heparine-Agarose. Five protein fractions displaying A_280_ (D1, D2, D3, D4, and D5) were obtained when the crude venom was applied onto the DEAE Sephacel column (2.0 cm × 12.0 cm), as shown in Figure 1(a). The pH used in this chromatographic step was 7.8, which is considered to be the optimum pH for the activity of most proteolytic enzymes already isolated from snake venoms [42–47]. The fibrinogenolytic activity was found in all the UV-absorbing fractions, but the highest activity was found in D4 fraction. This fraction was pooled, lyophilized, and used in the second step of purification on Sephadex G-75 column (1.0 cm × 120.0 cm), equilibrated with 0.05 M ammonium bicarbonate buffer, pH 7.8. In this step, D4 fraction was resolved in five fractions named D4G1, D4G2, D4G3, D4G4, and D4G5 (Figure 1(b)). 

The D4G2 fraction, which displayed fibrinolytic and azoproteolytic activities, was further purified on Heparine-Agarose Type I (2.0 cm × 10 cm) column, equilibrated with 0.02 M Tris-HCl buffer containing 5.0 mM CaCl_2_, and eluted with the same buffer until the tenth tube and then with 0.02 M Tris-HCl buffer containing 1.0 M NaCl. Two fractions (D4G2H1 and D4G2H2) were resolved (Figure 1(c)). D4G2H1 displayed fibrinogenolytic and azoproteolytic activities. Its specific activity was 657 U/mg, and this fraction was called Bmoo FIBMP-I, as shown in Table 1.

The protein yield, based on the absorbance at 280 nm, was 7.0 mg for D4G2H1 (Bmoo FIBMP-I) fraction. The enzyme represents 2.9% of the total protein in the venom (Table 1). The yield of Bmoo FIBMP-I was higher than those reported for other fibrinogenolytic enzymes purified from the same venom, for example, MSP1, MSP2 [15], and MOO3 [46] that yielded 0.51, 1.1, and 1.42%, respectively.

**Figure 2:**
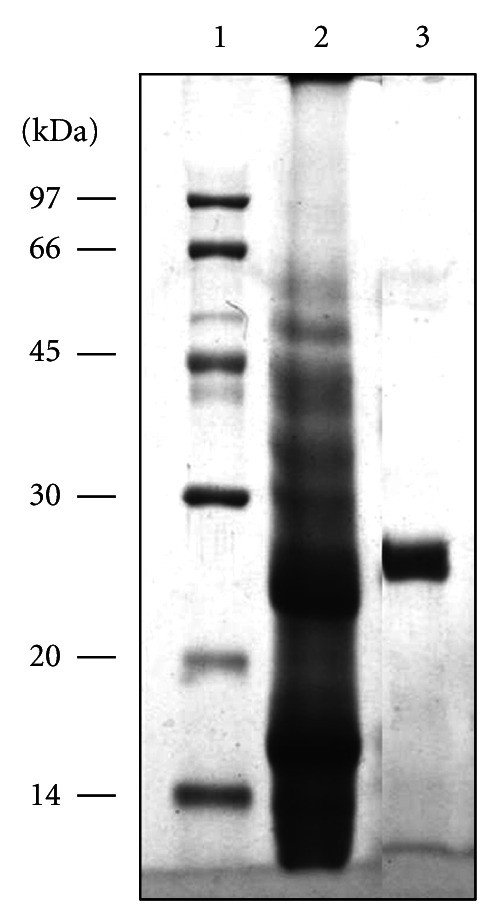
SDS PAGE electrophoresis of the crude venom and purified fibrinogenolytic enzyme from *B. moojeni* in 14% (w/v) polyacrylamide gel. Line 1: molecular mass standards. Line 2: reduced crude *B. moojeni* venom (50 *μ*g). Line 3: reduced Bmoo FIBMP-I (40 *μ*g).

Bmoo FIBMP-I was apparently homogeneous and migrated as a single band of a molecular mass estimated as 27.6 kDa by SDS-PAGE under reducing (Figure 2, lane 3) and native (data not shown) conditions. 

**Figure 3:**
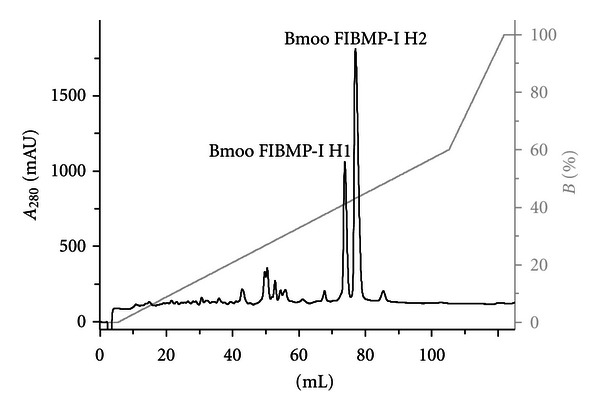
Reverse phase chromatography of Bmoo FIBMP-I. The fractions Bmoo FIBMP-I H1 and Bmoo FIBMP-I H2 did not show fibrinogenolytic or azocaseinolytic activities.

For best characterization, Bmoo FIBMP-I was applied onto an HPLC system using a reverse phase chromatography SOURCE column (0.46 cm × 25 cm), equilibrated with solution A (0.1% TFA in water), and eluted with a gradient of solutions A and B (0.1% TFA in acetonitrile). Two major fractions were obtained (Bmoo FIBMP-I H1 and Bmoo FIBMP-I H2) (Figure 3) that did not show fibrinogenolytic or azocaseinolytic activities, as expected. We believe that the loss of activity was due to the presence of the organic solvent acetonitrile. Both collected fractions were submitted to mass spectrometry analyses, showing molecular masses of 22.7 kDa and 22.9 kDa, respectively (data not shown). Both proteins are, most likely, isoforms with slight difference in their amino acid residues. The presence of protein isoforms is a common feature found in samples of proteases isolated from snake venoms [48, 49].

Bmoo FIBMP-I was resistant to cleavage by Edman N-terminal sequencing, suggesting that its N-terminal residue could be blocked by the presence of piroglutamic residue, a common feature in proteases from snake venoms, as demonstrated for a fibrinogenolytic protease of *Bothrops leucurus* venom described by Bello et al. [2] and confirmed by Ferreira et al. [50].

 The partial amino acid sequence (*de novo* sequencing) of Bmoo FIBMP-I was manually established by analyzing the MS/MS data in MALDI TOF TOF of peptides resulting from trypsin digestions (Table 2). As shown in Table 3, the data indicate that one of the tryptic fragments whose sequence is YIELVVVADHGMFTK (1,721 Da) shares homology with metalloproteinases of different snake venoms as, for example, a MP from *Bothrops moojeni*, BmooMP*α*-I [16], a MP from *Agkistrodon halys pallas* [51], and an hemorraghic factor from *Lachesis muta muta (Bushmaster)* [52].

The classes of MP from snake venoms are differentiated by the size and the presence of structural domains in addition to the metalloproteinase domain. These classes are named PI to PIII, with molecular masses ranging from 15 to 100 kDa [6, 53]. PI class contains exclusively the metalloproteinase domain and possess molecular mass around 25 kDa, with or without hemorrhagic effects [54]. It is divided into two subclasses: P-1A, formed by proteins as BthMP from *B. moojeni* venom [17], and P-1B, for example, Neuwidase from *B. neuwiedi* venom [19, 21]. Bmoo FIBMP-I showed high fibrinogenolytic and azoproteolytic activities, although its coagulant, anticoagulant, and haemorrhagic activities were absent. Thus, we suggest that Bmoo FIBMP-I is a PI-B class of metalloproteinase by considering its molecular mass of 22.8 kDa and the absence of hemorrhagic activity.

The structure of fibrinogen is composed of three pairs of disulphide-linked chains termed A*α*, B*β*, and  *γ*  chains. Following vascular injury, there are two principal mechanisms involving fibrinogen to control bleeding. It acts as an adhesive protein essential for platelet aggregation as well as forming an insoluble fibrin clot in the final stage of the blood coagulation cascade [55]. The action of proteinases that may interfere with coagulation and the fibrino(geno)lytic system following envenomation by *Bothrops* snake bites have been reported [53]. 

 The majority of the fibrinogenolytic enzymes in snake venoms are classified as “metzincins”, as described by Stocker et al. [56] and preferentially cleave the A*α*  chain of bovine fibrinogen with a slower cleavage of B*β*  chain [57, 58].

**Figure 4:**
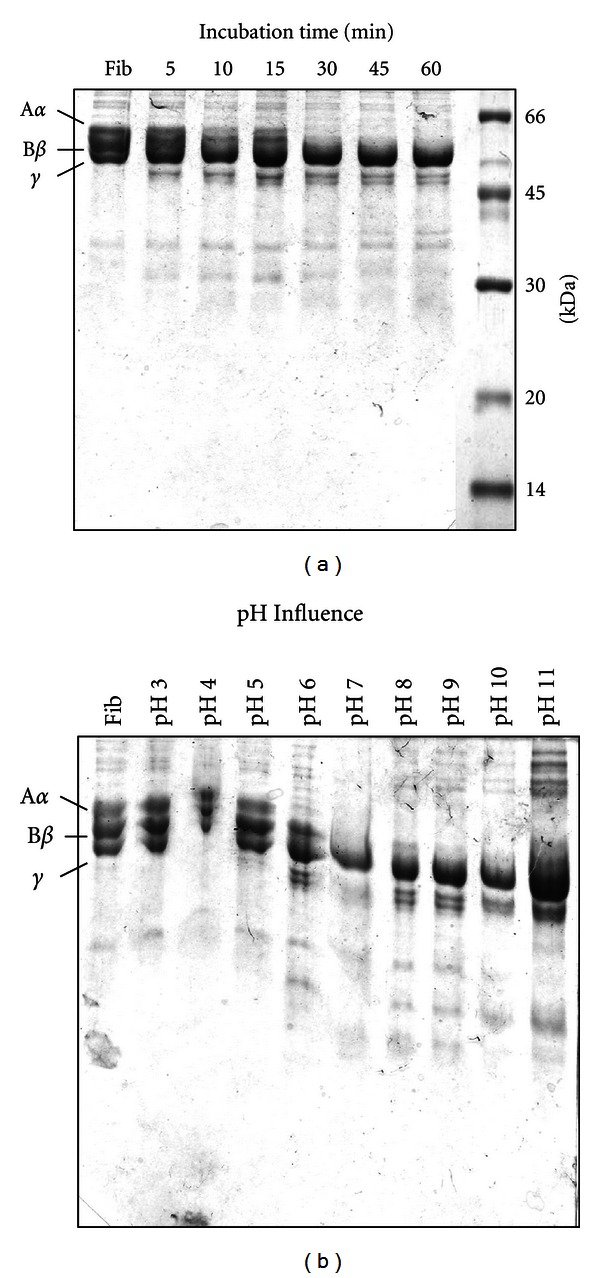
Fibrinogenolytic activities. (a) Bmoo FIBMP-I incubated with fibrinogen at different times (5, 10, 15, 30, 45, and 60 min), pH 7.8 at 37°C. Fibrinogen control incubated (without Bmoo FIBMP-I) for 60 min is showed in the lane on the left. Molecular mass standards are shown in the lane on the right. (b) Bmoo FIBMP-I incubated with fibrinogen at different pHs (3, 4, 5, 6, 7, 8, 9, 10, and 11), for 60 min at 37°C.

Bmoo FIBMP-I rapidly digested the A*α*-chain of bovine fibrinogen within 5 min, followed by B*β*-chain degradation, in 30 min, leaving the  *γ*  chain unaffected after 60 min of incubation (Figure 4(a)). Concomitant with the A*α*  and B*β*  chains partial digestion, degradation products were observed. Thus, the SDS-PAGE analysis (under reducing conditions) of this proteolytic process demonstrated that Bmoo FIBMP-I is an  *α*-fibrinogenase.

The optimal temperature (data not shown) and pH conditions for A*α*-chain digestion was 37°C and 6.0–10.0, respectively (Figure 4(b)). Hydrolysis of the A*α*  and B*β*  chains was partially inhibited by EDTA, a standard metalloproteinase inhibitor, and not by aprotinin, a serinoproteinase inhibitor (data not shown). This result confirmed that Bmoo FIBMP-I is a metalloproteinase, since it was inhibited by a chelating agent, such as EDTA.

The lack of blood coagulation observed in many envenomation circumstances is caused by the degradation of fibrinogen by fibrinogenolytic enzymes that cause defibrination* in vivo* [59]. Bmoo FIBMP-I degraded fibrinogen *in vitro*, although it did not prevent coagulation *in vivo*. This can be explained by the presence of endogenous inhibitors, which are able to neutralize the proteolytic activity of snake venom proteases, such as  *α*2-macroglobulin [59].

 Bmoo FIBMP-I can be compared to a nonhaemorrhagic enzyme named neuwidase identified in the *B. neuwiedi* venom by Rodrigues et al. [19] and Izidoro et al. [60]. This enzyme is described as a metalloproteinase with preferential activity towards the A*α*  chain of bovine fibrinogen and significantly less activity towards the B*β*  chain. Another fibrinogenolytic enzyme identified was brevilysin L6, from* Agkistrodon halys brevicaudus* snake venom [61, 62], which has a molecular weight of 22,713, with 203 amino acid residues. 

 Besides these fibrinogenases, an enzyme of 24 kDa, designated *Lachesis stenophrys* fibrinogenase was isolated from *Lachesis stenophrys* snake venom and classified as class PI metalloproteinase which displays fibrinogenolytic activity, but lacks haemorrhagic activity [4].

 Bmoo FIBMP-I can also be compared to the enzyme fibrolase, a zinc-dependent metalloproteinase, formed by a single chain of 23 kDa [63], with the N-terminal blocked by a pyroglutamate residue that is present in both isoforms [48]. This enzyme has preferential proteolytic activity towards the  *α*  chain of bovine fibrinogen (cleavage site between Lys^413^-Leu^414^ residues), a lower activity on the B*β*  chain [64] and no activity on the  *γ*  chain. Recent advances following prospective studies with snake venoms suggest a therapeutic application of fibrinogenolytic metalloproteinase in the treatment of occlusive thrombosis [65, 66]. Several of these enzymes have been studied *in vivo* in animal models and promising results have emerged [46, 65, 67–74].

Bmoo FIBMP-I showed proteolytic activity on azocasein and this activity was dependent on the pH and enzyme concentration, optimally occurring between pH 6–10.0 and being absent in acidic pH (3, 4, and 5) and alkaline pH (11.0). Considering our data and other tests of proteolysis using azocasein [39, 75, 76], one may conclude that the optimal pH for the proteolytic tests would be 8.8. Many proteases have the proteolytic activity on azocasein reduced, but not completely abolished, when incubated at pH 5.0, and these proteases have higher activity in alkaline pH and are inactive at acidic pH values [76, 77].

**Figure 5:**
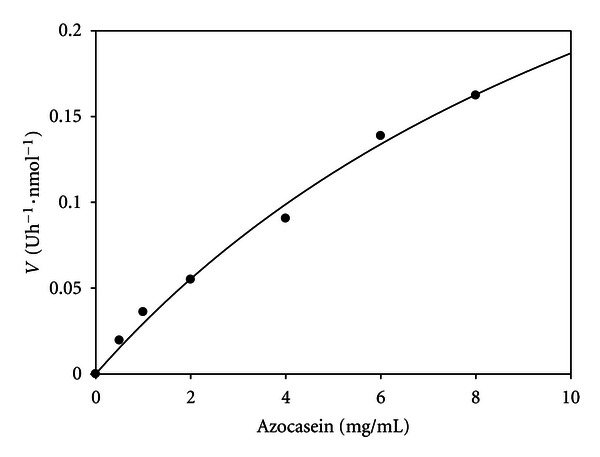
Michaelis-Menten plot of normalized velocity versus azocasein concentration. Each point represents the mean of two determinations. Statistic parameters: *r* ^2^ = 0.9931; *N* = 7; *P* < 0.0001.

 The proteolytic activity was assayed with different azocasein concentrations. The data were plotted as a Michaelis-Menten type plot (Figure 5), and the regression line (*r* ^2^ = 0.9931) lets us determine the *V*_max⁡_ = 0.4596 U h^−1^ nmoL^−1^ ± 0.1031 and *K*_*m*_ = 14.59 mg/mL ± 4.610. 

## 4. Conclusion

In conclusion, we have purified a metalloproteinase, named Bmoo FIBMP-I, with azocaseinolytic and fibrinogenolytic activities, from *B. moojeni* venom. The properties of Bmoo FIBMP-I indicate that this protein belongs to class PI-B of SVMPs. These features suggest that this toxin could interfere with the coagulation cascade that follows envenoming. The cleavage specificity of Bmoo FIBMP-I for fibrinogen raises the possibility of using this enzyme in formulations for clinical use.

## Figures and Tables

**Table 1:** Summary of the chromatographic steps of the Bmoo FIBMP-I enzyme from *Bothrops moojeni*.

Sample	Total protein (mg)	Protein recovery (%)	Total activity (units)*	Specific activity**
Crude venom	242.0	100	35,62	147
D4	27.38	11.31	8,310	303
D4G2	12.06	5 (44.04^1^)	4,544	377
Bmoo FIBMP-I	7	2.9 (58.03^2^)	4,601	657

*Proteolytic activity was tested with azocasein as substrate.

**One unit of specific activity was defined as units/mg protein.

^1^Protein recovery related to fraction D4.

^2^Protein recovery related to fraction D4G2.

**Table 2:** Sequencing *de novo* of Bmoo FIBMP-I peptides. Similarity searches were performed with the obtained sequences using the Fasta3 tool against the Swiss-Prot data Bank.

Peptide mass (Da)	Sequence
817,4044	NSINTLR
1175,4693	AYTGGMCDPR
1319,6585	TDQVNEDFVPR
1432,6530	ASGGQGGLELWSDR
1721,7799	YIELVVVADHGMFTK

**Table 3:** Similarities with the 1.72 kDa peptide from Bmoo FIBMP-I. AGKHP Metalloproteinase from *Agkistrodon halys pallas* (482 aa) (UNIPROT Q9PVK9_AGKHP [78]). LACMU hemorrhagic factor II from *Lachesis muta muta* (Bushmaster) (200 aa) (UNIPROT HRL2_LACMU [52]). Bmoo MP*α*-I Metaloproteinase from *Bothrops moojeni* (UNIPROT VM1BI_BOTMO [16]). AGKHB metaloproteinase (fragment) from *Agkistrodon halys* (UNIPROT Q90WC0_AGKHB [51]).

Protein	AA	Sequence															Similarity (%)	PDB
Bmoo FIBMP-I	—	Y	I	E	L	V	V	V	A	D	H	G	M	F	T	K		
AGKHP	198–212	Y	I	E	L	V	V	V	A	D	H	G	M	F	T	K	100	Q9PVK9
LACMU	5–19	Y	I	E	L	V	V	V	A	D	H	G	M	F	T	K	100	HRL2
Bmoo MP*α*-I	9–23	Y	I	E	L	V	V	V	A	D	H	G	M	F	K	K	97.8	VM1BI
AGKHB	33–47	Y	I	E	L	V	I	V	A	D	H	G	M	F	T	K	97.8	Q90WC0
